# Strengthening mental health and research training in Sub-Saharan Africa (SMART Africa): Uganda study protocol

**DOI:** 10.1186/s13063-018-2751-z

**Published:** 2018-08-06

**Authors:** Fred M. Ssewamala, Ozge Sensoy Bahar, Mary M. McKay, Kimberly Hoagwood, Keng-Yen Huang, Beverly Pringle

**Affiliations:** 1 Brown School, Washington University in St. Louis, 1 Brookings Drive, St. Louis, MO 63130 USA; 2 Department of Child and Adolescent Psychiatry, School of Medicine, New York University, 550 First Avenue, New York, NY 10016 USA; 3 National Institute of Mental Health, 6001 Executive Boulevard, Bethesda, MD 20892 USA

**Keywords:** Child mental health services, Multiple family group interventions, Evidence-based practice, Implementation science

## Abstract

**Background:**

Children in Sub-Saharan Africa (SSA) comprise half of the total regional population, yet existing mental health services are severely under-equipped to meet their needs. Although effective interventions for the treatment of disruptive behavioral disorders (DBDs) in youth have been tested in high-poverty and high-stress communities in developed countries, and are relevant for widespread dissemination in low- and middle-income countries (LMICs), most of these evidence-based practices (EBPs) have not been utilized in SSA, a region heavily impacted by poverty, diseases including HIV/AIDS, and violence. Thus, this paper presents a protocol for a scale-up longitudinal experimental study that uses a mixed-methods, hybrid type II, effectiveness implementation design to test the effectiveness of an EBP, called Multiple Family Group (MFG) aimed at improving child behavioral challenges in Uganda while concurrently examining the multi-level factors that influence uptake, implementation, sustainment, and youth outcomes.

**Methods:**

The MFG intervention will be implemented and tested via a longitudinal experimental study conducted across 30 public primary schools located in both semi-urban and rural communities. The schools will be randomly assigned to three study conditions (*n* = 10 per study condition): (1) MFG delivered by trained family peers; (2) MFG delivered by community health workers; or; (3) comparison: usual care comprising mental health care support materials, bolstered with school support materials. A total of 3000 children (ages 8 to 13 years; grades 2 to 7) and their caregivers (*N* = 3000 dyads); 60 parent peers, and 60 community health workers will be recruited. Each study condition will comprise of 1000 child-caregiver dyads. Data will be collected at baseline, 8 and 16 weeks, and 6-month follow-up.

**Discussion:**

This project is the first to test the effectiveness of the MFG intervention while concurrently examining multi-level factors that influence overall implementation of a family-based intervention provided in schools and aimed at reaching the large child population with mental health service needs in Uganda. Moreover, the study draws upon an EBP that has already been tested for delivery by parent peers and community facilitators, and hence will take advantage of the advancing science behind task-shifting. If successful, the project has great potential to address global child mental health needs.

**Trial registration:**

ClinicalTrials.gov, ID: NCT03081195. Registered on 16 March 2017.

**Electronic supplementary material:**

The online version of this article (10.1186/s13063-018-2751-z) contains supplementary material, which is available to authorized users.

## Background

Currently, an estimated 450 million people, most of whom live in poverty and are from low- and middle-income countries (LMICs), experience serious mental health challenges [1]. Children in Sub-Saharan Africa (SSA) comprise half of the total regional population, yet current mental health services are severely under-equipped to meet their needs [1, 2]. A recent systematic review estimated that one in seven children in SSA may struggle with a serious mental health issue. The World Health Organization (WHO) estimates prevalence rates may be even higher (20%) [3]. Uganda (one of the poorest countries in SSA) reports 12 to 29% of children presenting mental health symptoms when screened in primary care clinics [4, 5] and found that one in five Ugandan adolescents evidences a serious mental health challenge. Given the large numbers of children in Uganda, child disruptive behavior disorders (DBDs), if untreated, are a particularly serious concern as they commonly persist through adolescence and adulthood [6–9].

DBDs are chronic, impairing, and costly mental health problems that, when left untreated, carry a high price resulting from disruptions in school performance, friendships, and family relations. DBDs impair academic and social functioning, disrupt the parent-child relationship, are associated with child maltreatment and abuse, and commonly co-occur alongside depression, anxiety, and substance use disorders. The impairment associated with DBDs in childhood also place youth at increased risk for future school dropout, substance use/abuse, delinquency, incarceration, criminal behaviors, and premature death [6–9]. Studies have identified specific risk factors for increased incidence of DBDs among children, including poverty, low parental educational attainment, maternal depression, harsh parenting, poor parent-child relationship, stress, and orphanhood (the death of one or both parents) [10–13]. Studies emphasize adverse outcomes associated with DBDs, including academic problems, social impairment, a higher incidence of chronic physical problems, unemployment and legal problems, and substance abuse and violence among adults [9, 14–20].

Six SSA countries, namely Uganda, Nigeria, South Africa, Ethiopia, the Democratic Republic of Congo, and Kenya have DBD prevalence rates, ranging from 12 to 33% [21–24]. Given the serious consequences of failing to intervene as DBDs emerge, it is imperative that effective and scalable solutions are discovered, while simultaneously recognizing the challenges facing these countries in meeting the educational and mental health care needs of their large youth populations. In Uganda, the focus of this paper, children make up about half (56%) of the total population (compared to 20% in the US) [25], and they most often present with multiple simultaneous physical, mental health, and educational challenges [25, 26]. Ugandan children live in disadvantaged communities with high rates of chronic poverty (38%), domestic violence (30%), physical violence toward children (80%), depression (33 to 39%), malaria (70 to 80%), and HIV or AIDS (6%) [27–32]. The country also has a significant number of orphans [9, 30]. Hence, for DBDs to be addressed, poverty and family economic capacities, family and community safety, as well as health and mental health co-morbidities must be taken into account in any evidence-based practice (EBP) considered for scale-up.

### Treatments for DBDs

The basic principles underlying effective parenting practices and child development are considered cross-culturally robust [33–37]. Positive behavioral supports, effective behavioral management, positive parent-child relationship, and parent involvement in a child’s life play critically important roles in the healthy development of all children, regardless of cultural or ethnic background. Many DBD focused EBPs are designed to enhance parenting skills [38–41] via behavioral practice, modeling, coaching, goal setting, family communication, and building on family strengths [41–45]. These components are embedded within the MFG model. Although MFG and other similarly effective interventions for the treatment of DBDs in youth have been tested in developed countries and in similar high-poverty and high-stress communities, and they are relevant for widespread dissemination in LMICs, most of these EBPs are widely not used in SSA countries.

### Rationale for applying 4Rs and 2Ss MFG intervention for DBDs

Kazdin and Whitley [46] describe how specific family factors tied to poverty (e.g., stress) may undermine parenting (e.g., family organization, discipline practices, family connectedness, support, communication) and contribute to serious childhood behavior problems [47–49]. In collaboration with parents and service providers in the US, this body of research was summarized to encourage transparency of the evidence base for families and to provide an “easy to remember” means of organizing existing science for family peer MFG facilitators [50–54]. Specifically, four broad conceptual categories were created and became the family level targets for MFG: Rules, Responsibility, Relationships and Respectful communication (4Rs). Stress and Social support (2Ss) were added as these impact service engagement and outcome. This same review of the evidence regarding the influence of parenting, quality of family life and youth behavioral challenges was also conducted in South Africa as part of the CHAMP (Collaborative HIV prevention and Adolescent Mental Health Project), an EBP listed on SAMHSA’s National Registry of Evidence-based Programs and Practices.

The MFG (guided by the 4Rs and 2Ss) involves six to eight families in the US and 12 to 20 families in South Africa [40, 55–63]. At least two generations of a family are present in each session. Content and practice activities foster both within-family and between-family learning and interaction [64]. The MFG targets primary school age children aged 7 up to 13 years in the US and South Africa. In the US, the MFG is designed for children struggling behaviorally and their families. In South Africa, MFG has targeted youth at high contextual risk for behavioral challenges (high rates of poverty, violence, family loss due to HIV and other health threats). In both countries, children and their families (including adult caregivers and siblings over 6 years of age) are invited to attend 16 sessions.

MFGs are also the core of SUUBI-Maka (Hope for families in Luganda), a family focused, economic-strengthening program tested in Uganda, [10–13, 65–71] furthering the evidence for their feasibility and acceptability in the specific context of this scale-up study. In addition, the development of MFG was guided by recommendations offered by Weisz [72] and Hoagwood [73] who argued that to improve implementation of EBPs, development and testing must occur in the setting where the service will ultimately be embedded, with the prior work in South Africa (SA) giving the MFG an important head start in learning about factors that influence implementation and scaling [40, 61, 70, 74, 75]. Also, both in the US and South Africa, MFGs have been facilitated by lay counselors or community health workers/village health teams, and parent peers, consistent with task-shifting strategies.

### Contextual influences on implementation and scale-up

**Fig. 1:**
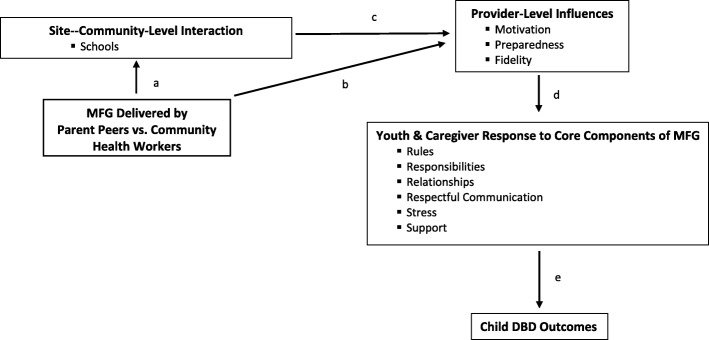
Multi-level influences on Multiple Family Group (MFG) implementation and child outcomes

Adapting EBPs to SSA countries requires thoughtful consideration of individual- and system-level factors [76–79]. Interventions developed in academic isolation too often fail to address the real-world constraints of settings in which they will be used – insufficient resources, limited workforce capacity, and failure to partner with funders and policy-makers [80]. In our previous work and in the current study, we draw upon PRISM (Practical, Robust Implementation, and Sustainability Model). PRISM is a practical and comprehensive implementation framework that integrates aspects of diffusion of innovation, models for quality improvement, and Reach-Effectiveness-Adoption-Implementation-Maintenance (RE-AIM). PRISM emphasizes: (1) organizational perspectives on an intervention (e.g., feasibility, adaptability, barriers); (2) external environment (e.g., community resources); (3) recipients’ characteristics (provider and parent response); (4) implementation and sustainability infrastructure (training and supervision supports); and (5) the RE-AIM approach to scaling. PRISM provides a framework to study the interaction of interventions with the characteristics of multi-level contexts/factors which may influence uptake, implementation, integration and youth outcomes (youth and adult caregiver response, provider preparedness, motivation and fidelity, community-level support). Figure 1 summarizes how site- (schools) and provider- (parent peers and community outreach health worker (CHW)-level variables may reflect key influences on implementation and ultimately child outcomes of MFG (not shown in this diagram is the contrast between the MFG and the comparison condition, which reflect the ultimate impact of the two MFG variants on child outcomes, these analyses will also be pursued). The delivery of MFG by parent peers versus CHWs is assumed to impact site (schools) and community-level interaction (path a) and provider-level influences (path b). Site and community-level interaction, in turn, affects provider-level (facilitators) variables (path c). These differential effects then carry through to the core family membes (path d), and in turn, child outcomes (path e).

Thus, in this paper, we present and describe in detail a protocol for a scale-up longitudinal experimental study that uses a mixed-methods, hybrid type II, effectiveness implementation design [81] to test the effectiveness of an EBP, called the Multiple Family Group (MFG) family strengthening intervention, aimed at improving child disruptive behavioral challenges in Uganda while concurrently examining implementation – specifically, the multi-level factors that influence uptake, implementation, sustainment, and youth outcomes. In summary, the objective of the proposed study is to examine the implementation and outcomes associated with an EBP, specifically MFG targeting youth disruptive behavior challenges and success, through a scale-up intervention study in Uganda (see https://sites.wustl.edu/smartafrica/ for more details about the SMART Africa Center). More specifically, the study objectives are:

1. To examine short-term and longitudinal outcomes associated with the MFG (disruptive child behavior, behavioral functioning)
2. To compare the uptake and implementation of MFGs by trained existing family peers and CHWs
3. To elucidate multi-level factors that influences uptake, implementation and youth outcomes

## Methods

**Fig. 2:**
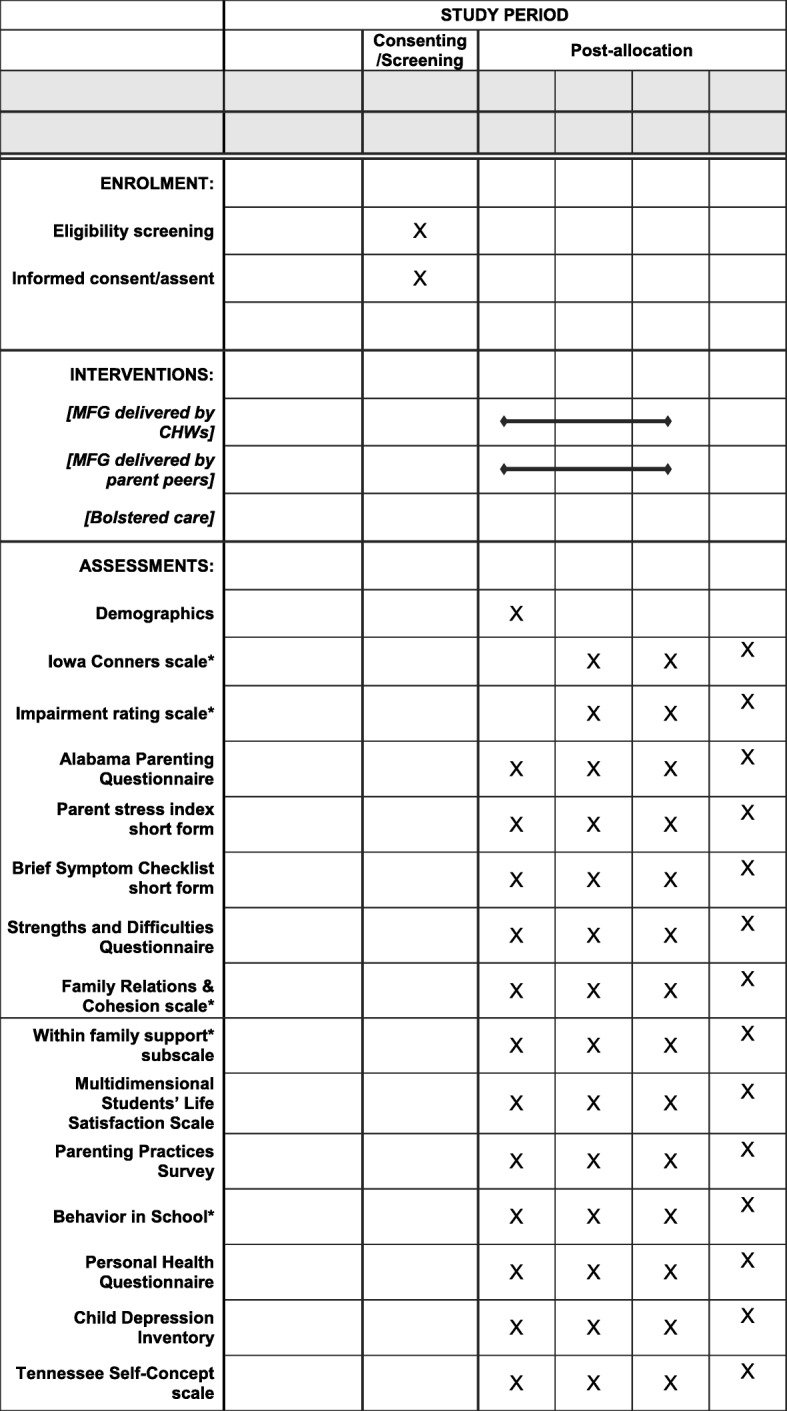
Standard Protocol Items; Recommendations for Interventional Trials (SPIRIT) schedule of enrollment, interventions, and assessments

The proposed study will be conducted across primary schools representing both semi-urban and rural communities. We expect to involve 3000 youths (in primary schools grades 2 through 7; 8 to 13 years) and their adult caregivers (3000) in Uganda. Schools will be randomly assigned to three study conditions: (1) MFG delivered by trained family peers; (2) MFG delivered by CHWs; or, (3) comparison: mental health materials. Data will be collected at baseline, 8 and 16 weeks, and 6-month follow-up (10 months from baseline; see Fig. 2 for the Standard Protocol Items: Recommendations for Interventional Trials (SPIRIT) schedule of enrollment, interventions, and assessments). A SPIRIT checklist (see Additional file 1) has also been included for further details on the study protocol.

### Study setting

The proposed study will target youth attending primary schools in Rakai District and the greater Masaka region (southwestern Uganda) as well as their caregivers. The region is heavily affected by poverty with high HIV prevalence rates (at least 8.5% vs. 6.5% found in the national average) [82]. The area also has large number of children with no living biological father, mother or both (also known as orphaned children). An estimated two in five children are orphaned to AIDS each year in these districts [82].

### Study population, recruitment, and retention

#### Sampling and recruitment

##### Schools

The study involves the active collaboration of the Masaka Diocese which operates slightly over 420 public primary schools in Rakai District and the greater Masaka region. We will recruit medium-sized schools to participate in the study. All schools with a total enrollment of more than 900 students will be eliminated as they are considered above average enrollment. Similarly, all schools with a total enrollment of less than 350 students will be dropped because they are considered below the average normal enrollment of at least 50 students per grade. Schools that remain on the list after this stratification will be contacted to schedule informational meetings with the school leadership and staff (school heads/directors, head teachers, and teachers) to present the research study and gauge interest in study participation. Follow-up meetings will be scheduled when necessary. The research team will compile a list of the schools that express commitment to participating in the study (see the “Randomization” section for further details).

##### Children and caregivers

Schools will be asked to organize meetings for all families to learn about the study. All research staff will be on hand at each meeting to provide the opportunity for parents to ask questions about the study individually or as part of a larger group of parents. If parents and children are interested in participating, each of them will have an opportunity to meet individually with research staff and provide informed consent and child assent.

Following consent procedures, parents will be asked to complete screening tools for child DBDs (see Table 3). The screening will be conducted by project coordinators and research assistants. If the child meets the criteria for a clinically meaningful behavioral problem on any of the screening measures, they will be considered “positive”. They and their caregivers will be invited to enroll in the study and an appointment to complete a full baseline assessment (for both child and caregiver; see the “Measures” section) will be made prior to beginning to attend MFG meetings. If a child does not evidence a DBD, they will be considered “negative.” The family will be enrolled to attend the MFG meetings (all families in the school community will have the opportunity to attend to decrease stigma, but also enhance functioning of all parents/families). An appointment to complete a shortened version of the baseline assessment (for both child and caregiver; see the “Measures” section) will be made prior to beginning to attend MFG meetings. The child and the caregiver will be monitored at each time point with shortened versions of the assessment batteries. If at any point during these assessments, the child meets criteria for DBD, both the child and the caregiver will complete full version of the assessment batteries.

School meetings is the first way that we will reach out to parents of children enrolled in the potential schools. Research staff will also conduct home visits (as has been typically done in other studies in the area). In total, we will recruit 3000 children and their adult caregivers across three study conditions. We will continuously monitor study participants’ recruitment and enrollment during weekly staff meetings to ensure equivalent involvement across conditions by: gender of youth, age and adjust if imbalance occurs accordingly.

##### Parent peers and CHWs to facilitate the adapted MFG

At each of the school sites assigned to the parent-peer-delivered condition (*n* = 10), the head of school and the Chair of the Parents Teachers Association (PTA) will identify up to six parent peers to receive the training to deliver the adapted MFG. Parent peers will have already received some training for their current roles (e.g., workshops in methods to reach out to parents whose children are not performing well in school or not attending; facilitation skills in relation to organizing parent meetings). We expect to recruit 60 parent peers from the 10 schools in the parent-delivered condition.

At each of the school sites assigned to the CHW-delivered condition (*n* = 10), we will collaborate with the village local leaders, the district health officials, and the Ministry of Health (MOH) to identify up to 60 existing outreach workers associated with local primary care clinics in the study area. CHWs have already received training in health assessment, home and school outreach and facilitation of health-related meetings. Many of the CHWs are also local leaders within their communities. We expect to recruit 60 community health outreach workers to deliver the adapted MFG to children from 10 schools assigned to their condition.

##### Inclusion/exclusion criteria

Inclusion criteria are as follows: (1) school head teacher willing to participate; (2) parent peers or CHWs willing to participate; (3) adult caregiver of a child in primary 2 through 7, 8 to 13 years, willing to consent and available for research and intervention activities; (4) a child screened for oppositional defiant disorder (ODD) or conduct disorder (CD) and willing to assent. Exclusion criteria include lack of understanding of the study and participant rights and refusal to participate. If the youth or adult caregiver presents with emergency needs (e.g., hospitalization), needed care will be secured, rather than study participation.

#### Sample attrition and retention

We believe that attrition will be minimal because the investigative team is known in the schools and communities, and has substantial experience recruiting and tracking “hard to reach” families. The study will take place in a highly stable region of Uganda, where geographical moves are rare. Using the same protocols that we have used in our prior studies, we will ask the participants to give their postal box number and telephone number (if they have one), and names, addresses, and contact information for three people who will always know how to reach them. Participants will be reminded that if we are to contact the people listed, we would never discuss any details about their involvement in the study. We will use these records to track the location of study participants over time. We have effectively used these methods in other research programs, resulting in very low attrition rates (for SUUBI-Uganda, 2.4% at 10-month follow-up and 7.3% at 24-month follow-up; Suubi+Adherence, 4.3% at 48 months, and Bridges, 4.1% at 12 months, 9.3% at 48 months) [10–13, 65–71]. Hence, we expect attrition from baseline to the end of follow-up to be no more than 20%. We will keep careful records for those who drop out of the study and test for attrition bias based on data that we will have prior to study dropout. To the extent that such bias is present, we will limit generalizations accordingly, or where possible, introduce statistical adjustments (hot deck imputation methods [83–86] to complete missing observations, and thus to address bias.

#### Statistical power calculations

The present research uses multi-level structural equation modeling (MSEM) as opposed to traditional multi-level modeling (MLM) because of the presence of mediation [87]. The two are closely related, but the former is primarily available in the Mplus computer software. Power computations in MSEM models are complex because of the large number of assumptions about population parameters that must be made [87–89]. One approach is to power the study based on limited information estimation strategies that focuses on the most sample size demanding equation in the broader system of equations [90]. For longitudinal MLM studies, this also requires taking into account the potential presence of correlated errors of measurements, person-specific effects, and dropout [91–95]. Hedeker and Gibbons [96] also provide a power analysis approach that allows for specification of anticipated attrition patterns and correlations among the repeated observations. Power analyses for a linear trend over time take into account: (1) four measurement time points; (2) a three-group design; (3) a random effect for time and a random effect for facilitator within school for provider-type hypotheses; (4) a residual autocorrelation of rho = .3 and; and (5) an 8% attrition rate between successive time points. Randomization is at the school level. For the hypotheses involving children nested within facilitators or schools, our sample size calculations also incorporate recommendations outlined in Donner et al. [97], and allow for an inflation factor such that our effective sample size is two thirds that of the actual sample size. Table 1 presents detectable effect size (a between group difference increasing linearly from 0 at baseline to effect size measured in standard deviation (SD) units at the last time point) corresponding to 80% power.

**Table 1:** Power analysis

Unit (*n* per group)	4 time points	Effect size
School (*n* = 30)	0.39	moderate
Facilitators (*n* = 120)	0.28	moderate
Family (*n* = 3000)	0.10	small

### Study conditions

#### Treatment arms

The MFG platform for EBP delivery is a series of weekly meetings guided by a protocol [56, 60, 75]. Groups are held weekly and are facilitated by trained and supervised group leaders (in this case either parent peers or community health outreach workers). Groups can consist of up to 20 families involving adult caregivers and all children over 6 years of age in the family [74, 75, 98]. MFG targets, family skills, and goals for the manual to be used in Uganda—adapted from the 4Rs and 2Ss in the US, CHAMP South Africa and Suubi+Maka (Uganda)—are summarized in Table 2.

**Table 2:** Multiple Family Group (MFG) targets, family skills, and goals

MFG target	Empirically supported family skill	MFG goals
*R*ules	Family organization; consistent discipline	Clarify rules, consequences, rewards
*R*esponsibility	Inter-connectedness; expectancies	Clarify responsibilities, expectations, rewards
*R*elationships	Family warmth; within-family support	Schedule for positive family interaction
*R*espectful communication	Family communication; family conflict	Listening/ talking skills (parents/children)
*S*tress	Parenting hassles and stress; life events	Identify stressors undermining family change
*S*ocial support	Social isolation	Within-family and external support plan

#### Description of MFG intervention protocol

The protocols have been designed to provide multiple opportunities during each session to directly apply content to the realities of family life, emergent cultural and values perspectives, as well as to tailor messages to age of child. We have built in redundancy for missed appointments and opportunities for reinforcement. We aim for families to attend at least seven to eight meetings, as findings suggest that this dose is needed for reductions in child behavioral difficulties and prior studies in the US and SA suggest that most families will reach this goal [57, 58].

#### Program delivery

We will train at least six facilitators per school site to deliver the MFGs in both MFG-parent-peer-delivered and MFG-CHW-delivered condition. Training for parent peers and CHWs will occur separately, by condition. Incentives to participate and complete training will include: receiving certification in child mental health competency to be offered by the local district leaders—including the LC5 Chairperson and the district health official, and transport facilitation needed to complete project tasks and a community-wide recognition event thanking facilitators for their contributions.

#### Training and supervision

Training will consist of up to six modules. Training focuses on childhood conduct difficulties, family-level factors that have been linked to child outcomes, strategies to enhance engagement and motivation, and group facilitation skills and processes specific to MFGs. At the end of the MFG training, we will administer a Knowledge and Skills Assessment Test (KSAT), to assess mastery of the content (live competence demonstrations and knowledge questions read aloud to facilitators). The criteria of mastery for the KSAT will be set at 85%. During the MFG implementation period, facilitators will receive 2 h per month of group supervision across sites while the MFGs are in progress.

#### Description of the comparison condition

Mental health wellness materials from the MOH will be made available to all children and families recruited in the study (across all the three study conditions). We will adapt printed materials on child mental health based on those recently created by the MOH.

#### Adaptation of MFG manual

During phase I (months 1 to 12), the study team will review the existing MFG intervention protocol, as well as the South African training and delivery methods used in the CHAMP program of research (e.g., cartoon format of information delivery and family practice activities, training protocols for lay counselors and community facilitators), and the SUUBI family economic strengthening approach. Further, these working groups will review the existing evidence related to the relationship between specific parent management practices, family organizational processes and livelihood approaches, and positive youth behavioral outcomes in order to align with perspectives and organization of families in SSA. The intervention adaptation team will comprise the in-country research team as well as community stakeholder volunteers (one to two teachers, one to two parents, one to two CHWs) recruited to assist with intervention adaptation.

In the Ugandan context, faith-based institutions, including the church and religious leaders, are longstanding partners to the investigative team. The same guidelines for partnerships that organize our work with local, regional and national policy-makers, and non-governmental organizations (NGOs) also guide our intensive collaboration with religious, cultural, and community leaders. These partnerships are characterized by intensive, ongoing involvement and communication with substantial effort invested in developing shared goals, understanding and feedback processes across the entire span of the project and country contexts.

The adaptation process will follow steps previously outlined by Petersen and McKay [40, 61, 75]. Specifically, interaction between in-country and cross-country teams will be intensive and systematic. Team members will identify core elements of the existing science on parenting and family life that need to be addressed within each country context (for example, in prior studies set in SA, rapid ethnographic studies were conducted to identify elements of family life that were missing from existing evidence-based, particularly perceptions of disempowerment of parents, harsh discipline strategies, parents’ and children’s rights) and incorporate evidence-informed intervention elements into the adapted MFG intervention and training protocols.

### Randomization

Stratified cluster randomization of schools to conditions will be used, with schools stratified into four strata based on two variables: (1) student population size (medium size vs. large) and; (2) geographical location (rural vs. semi-urban), to ensure balance on those variables. The restricted randomization technique of Hayes and Moulton [99] will be used to assure overall school balance across the three experimental groups. Each school will be randomly assigned to one of the three study conditions such that all selected children from a particular school will receive the same intervention (reduce contamination). These schools are geographically apart, with limited transportation. Hence, the risk for contamination will be minimal (See the “Contamination” section).

Specifically, to select and randomize the potential schools, we will follow the following procedure:

1. A list of the primary schools in the area will be obtained from the Masaka Diocese that oversees all the 420 church-founded but government-supported public primary schools in the Rakai District and Masaka regions; and the District Education Office
2. All schools with a total enrollment of more than 900 students will be eliminated as they are considered above average enrollment. Similarly, all schools with a total enrollment of less than 350 students will be dropped because they are considered below the average normal enrollment of at least 50 students per grade
3. Schools that remain on the list after this stratification will be contacted to schedule informational meetings with the school leadership and staff (school head teachers, and teachers) to present the research study and gauge interest in the study. During these meetings, the purpose of the study will be explained and what the school’s participation entails will be laid out. All study-related questions will be answered. Follow-up meetings will be scheduled when necessary
4. The research team will compile a list of the schools that express commitment to participating in the study
5. Thirty schools from this list will be randomly chosen
6. Using SPSS software version 24, each of these 30 schools will be randomly assigned to one of the three study conditions, i.e., 10 to the adapted MFG delivered by parent peers; 10 to the adapted MFG delivered by CHWs; and 10 to the comparison condition

We estimate that a total of 30 schools will allow us to identify sufficient number of youth and nest them within trained program facilitators. This will allow the research team to detect a small to moderate effect based upon power calculations. Thirty schools is our best estimate of the total student population needed to draw a sufficient sample. The number of schools will be adjusted if needed.

### Contamination

Because the experimental-control manipulations are applied across schools that are a considerable distance from each other, and CHWs and parent peers have their own unique designated site, we expect contamination to be minimal. Specifically, all children from the same school and their families will be involved in the same study condition. The distribution of materials is unlikely to influence implementation given that the schools are geographically separated, with limited transportation opportunities in between schools. However, the in-country research team will monitor the extent of any such “seepage” and make statistical adjustments for it, if needed. Monitoring will be established by directly asking facilitators whether any intervention materials were shared with staff/caregivers from other schools every time they complete the delivery of a 16-week intervention and their answers will be carefully documented by the research team (including when, how many people, which materials, and which schools, when relevant). More specifically, this assessment will take place when the research team is in the field to complete the 16-week assessment with caregivers. Again, we do not anticipate contamination given the limited interaction across schools.

### Data collection

Multiple sources of data collection, including Facilitator (F), Caregiver (C), Child (Ch), Research staff (R), and School head (SH) reports, will be used. Following screening (S), data will be collected baseline (T1), 8 weeks (T2; mid intervention) and 16 weeks (T3; post intervention), and 6-month follow-up (T4). All caregiver and child assessments will be available in English and Lugandan (translated using rigorous forward/backward methods). Depending on the existing health surveillance system and resources under the Ministry of Education (MOE) or Ministry of Health (MOH), we will work with ministry stakeholders prior to the scale-up study (during the first 18 months of the capacity-building stage) to determine options to adapt the EBP monitoring systems into school or community service settings. The measurement strategy will be guided by the RE-AIM evaluation framework [100, 101]. The RE-AIM conceptualizes the public health impact of an intervention as a function of five dimensions— reach, effectiveness, adoption, implementation, and maintenance. *Reach*; target populations (CHWs/parent peers, families attending MGFs) will be characterized in terms of demographics, motivation, and child behavior. *Effectiveness*: we will evaluate the impact of MFGs on facilitators, parents, and children. *Adoption:* to characterize the target setting (school/ community clinic), data will be collected at baseline from two sources. *Implementation*; to determine the extent to which the MFG is implemented as planned, checklist-based fidelity monitoring systems have already been designed based on ground breaking work of Schoenwald [102, 103]. Research staff will be trained to used fidelity tools that tap multiple dimensions of a MFG session, including presence and quality of facilitation related to the following: (1) session content; (2) group discussion; (3) use of activities to foster information exchange across families; (4) practice activities within families; (5) summary of learning; (6) explanation of homework; and (7) summary of family strengths. Independent fidelity observations will be conducted by research staff for 20% of MFGs. These data will be used to assess the relationship between planned and actual implementation, to evaluate the integrity of the implementation and how strategies were altered to maximize effectiveness and acceptability. *Maintenance/Sustainment*: We consider three aspects of maintenance: (1) *Implementation maintenance*: we will evaluate CHWs’ and parent peers’ ability to implementing the EBP over time. Facilitators practices during the subsequent implementation cycles (with different groups of families/students) will be measured in each cycle over 2 years via self-report and fidelity observations; (2) *Examine factors that may influence maintenance*: we will systematically examine individual, system level, and other contextual factors that may influence maintenance of the EBP; (3) *System-level sustainability*: to examine sustainability of the EBP within the organizations/sites (from both financial and policy perspectives), we will conduct select qualitative interviews with purposively selected MFG facilitators (*n* = 15 parent peers and *n* = 15 CHWs), school head teachers (*n* = 10) and in-country team members at four time points—baseline, 8 and 16 weeks, and 6-month follow-up. Informants will be purposefully sampled to reflect diversity of schools and perceived level of success in achieving implementation and sustainment of MFGs. Purposeful sampling is used to achieve maximum likelihood of achieving depth of understanding of patterns and processes of implementation and sustainment [104]. Interview guides will consist of semi-structured questions relating to experience with implementing and integration the MFG [105, 106]. Participants will also be asked to provide a narrative account of efforts to implement the MFG, including barriers and facilitators experienced. Table 3 lists the quantitative data collection tools, constructs, informant, and timing.

**Table 3:** Measures

Instruments	Reporter	Timing
Aim 1. To examine the short- (8 and 16 weeks) and long-term (6-month) outcomes (primary outcomes) associated with the Multiple Family Group (MFG)		
1. Disruptive Behavior Disorder Rating Scale [121]	C	S
2. Iowa Connors and Impairment Scales [122, 123]	C	S, T2, T3, T4
3. Alabama Parenting Questionnaire (APQ)-short form [124]	C, Ch	T1, T2, T3, T4
4. Parent Stress Index-short form [125]	C	T1, T2, T3, T4
5. Brief Symptom Checklist-short form [126]	C	T1, T2, T3, T4
6. Strengths and Difficulties Questionnaire [127]	C	T1, T2, T3, T4
7. Family Relations and Cohesion scale (adapted from Parent Child Relationship Inventory, Child Caregiver Communication Scale, Family Assessment Measure, and Family Adaptability and Cohesion Scale) [10–13, 65–71]	C	T1, T2, T3, T4
8. Within-family support subscale from Family Assessment Measure [10–13, 65–71]	Ch	T1, T2, T3, T4
9. Multidimensional Students’ Life Satisfaction Scale [128]	Ch	T1, T2, T3, T4
10. Parenting Practices Survey (PPS) [10–13, 65–71]	Ch	T1, T2, T3, T4
11. Behavior in School [10–13, 65–71]	C	T1, T2, T3, T4
12. Personal Health Questionnaire [10–13, 65–71]	Ch	T1, T2, T3, T4
13. Child Depression Inventory (CDI-short form) [129]	Ch	T1, T2, T3, T4
14. Tennessee Self-Concept Scale [130]	Ch	
Aim 2. To examine the uptake, implementation, fidelity, and sustainment of two MFG implementation approaches.		
4. Knowledge, Skill, Attitude Test (KSAT) for MFG facilitators [131]	F	T1
5. Attendance logs	F	every MFG session
6. MFG Intervention Fidelity Assessment [131]	F, C, R	every MFG session for F & C, every other session for R
7. Implementation and Feasibility Assessment [131]	F, SH	T2, T3, T4 for F; T3 for SH
8. Program Sustainability Assessment [132]	F, SH	T2, T3, T4 for F; T3 for SH
Aim 3. To elucidate multi-level factors that influences uptake, implementation, sustainment, and youth outcomes.		
MACS process measures [131]	F	T2, T3, T4
Qualitative interviews	F, SH	T3

Key: Facilitator (F), Caregiver (C), Child (Ch), Research staff (R), and School head (SH)

Screening (S), baseline (T1), 8 weeks (T2; mid intervention), 16 weeks (T3; post intervention), and 6-month follow-up (T4)

### Data analysis plan

#### Qualitative data analysis

All qualitative interviews will be digitally recorded and transcribed. Interviewers will then compare transcripts with digital records to insure accuracy of transcription. All field notes, interview transcripts, and interviewer notes summarizing interviews will be entered into NVivo [107]. Accuracy of information will be assessed through triangulation in which accounts of specific events and behaviors obtained from field notes, interviews, surveys, and archival material are compared [108]. To insure credibility of findings and enhance the validity and reliability of data, all interviews will be reviewed by at least two members of the research team. Consensus on coding and coding procedures and modifications to coding books will occur through regular team meetings. Study results will be presented to informants, enabling them to provide comment of results and suggest modifications or additional avenues of investigation.

Using a methodology of “Coding Consensus, Co-occurrence, and Comparison” [109], implementation team logs and interview transcripts will be analyzed in the following manner. Each investigator will review this material to develop a broad understanding of content as it relates to the project’s specific aims and to identify topics of discussion and observation. Investigators will prepare short descriptive statements or “memos” to document initial impressions of topics and themes and their relationships and to define the boundaries of specific codes (i.e., the inclusion and exclusion criteria for assigning a specific code) [110]. The empirical material contained in the field notes and interview transcripts will then be independently coded by the project investigators to condense the data into analyzable units. Segments of text ranging from a phrase to several paragraphs will be assigned codes based on a priori (i.e., from the interview guide) or emergent themes (also known as open coding) [111]. Following the open-coding, codes will be assigned to describe connections between categories and between categories and subcategories (also known as axial coding) [111]. Codes will also be assigned to material to reflect the social and demographic characteristics of study participants. Lists of codes developed by each investigator will be matched and integrated into a single codebook.

Five transcripts will be independently coded by at least two investigators. Disagreements in assignment or description of codes will be resolved through discussion between investigators and enhanced definition of codes. The final list of codes or codebook, constructed through a consensus of team members, will consist of a numbered list of themes, issues, accounts of behaviors, and opinions that relate to coalition structure, function, development, and sustainment. With the final coding structure, two investigators will separately review a sample of transcripts to determine level of agreement in the codes applied. A level of agreement ranging from 66 to 97%, depending on level of coding (general, intermediate, specific), indicates good reliability in qualitative research [112].

Upon completion of the coding of the remaining transcripts, NVivo will be used to generate a series of categories arranged in a treelike structure connecting text segments grouped into separate categories of codes or “nodes.” These nodes and trees will be used to further the process of axial or pattern coding to examine the association between different a priori and emergent categories. They will also be used in selective coding of material to identify the existence of new, previously unrecognized categories. The number of times these categories occur together, either as duplicate codes assigned to the same text or as codes assigned to adjacent texts in the same conversation, will be recorded, and specific examples of co-occurrence illustrated with transcripts. Through the process of constantly comparing categories with each other, the different categories will be further condensed into broad themes.

#### Quantitative data management and analyses

1. *Exploratory scale structure analysis*. The research will use robust estimators (in the form of Huber-White estimation) to accommodate non-normality and variance heterogeneity. We will conduct formal tests of outliers and be sensitive to specification error. Inter-item reliability analyses for all scales will be conducted using confirmatory factor analytic (CFA) perspectives; correlations across time and construct will be examined
2. *Data-reduction procedures*. Most of the proposed instruments have summary scoring procedures; however, multiple scores in specific domains may result in multicollinearity, requiring further data reduction. Various data-reduction strategies will be conducted, including factor analyses with oblique rotation [113] and Chi-squared Automatic Interaction Detector (CHAID) algorithms, to identify the strongest variables in the pool. The Exhaustive CHAID method, developed by Biggs, deVille and Suen [114] will be used to explore various groups of predictor variables as they relate to outcomes
3. *Tests of sample equivalence across study conditions*. We will use baseline data to examine potential biases related to possible attrition by condition or selection biases not addressed by random assignment. Preliminary analyses will be performed to compare the schools, facilitators, and adult caregivers in the three study conditions on descriptive/clinical characteristics at baseline to ensure that randomization succeeded. Any ancillary variables that are not the focus of hypotheses, but relate meaningfully to outcomes within treatment groups are candidates for inclusion as covariates. When covariates differentiate treatment groups, they will be included in all analyses to remove confounding effects
4. *General statistical considerations*. To address *aims # 1 and 2*, we will use random-effects models to examine scores over the four assessments. Random-effects models have characteristics that provide solutions to commonly observed problems in the analysis of longitudinal data; missing data, serial correlation, and time-varying covariates [114]. These analyses can model systematic person-specific deviations from the average time trend. Just as the nesting of participants within providers is an example of multi-level data, the nesting of perspectives within teacher reports can also be viewed as multi-level data. By treating treatment perspective as a “repeated measurement” we can estimate the difference between the three conditions on outcomes as a synthesis of the three perspectives. Divergence between the perspectives can also be examined by including interactions between covariates, condition and the rater effect. We will model each outcome as a function of group, time and group-by-time interaction. We will use Supermix software for the mixed-effects linear regression [96].
  To address aim 3, our analyses will use variants of structural equation modeling (SEM) with Mplus. The models we test often will be statistically over-identified. We will evaluate model fit using both global fit indices (e.g., relative/normed chi-square *χ*^2^/df with range 2.0–5.0) [115, 116], comparative fit index (CFI) (with value ≥ 0.95) [117], standardized root mean square residual (RMR (below 0.08) [117], root mean square error of approximation (RMSEA) (with the lower limit is close to 0 and the upper limit less than 0.07) [118] and indices of fit that are diagnostic of specific points of ill fit in the model (e.g., standardized residuals, modification indices). If poor model fit, then we will examine diagnostics that suggest revisions to the model that are meaningful and that will significantly improve model fit.
5. Hypotheses testing

### Aim 1. To examine short-term and longitudinal behavioral outcomes associated with the MFG

#### Hypothesis

Children who participate in MFG with their families will display significantly reduced conduct difficulties and increased functioning over time compared to those involved in the comparison condition. We expect that parent peers will evidence significantly more success engaging families to attend MFG sessions; thus, children in the MFG-parent-peer-delivered condition will evidence greater improvement relative to the other two study conditions.

We will consider a random-effects model for the child outcome variables across time as a function of treatment group, time and the interaction of group by time. A number of features will be common to all models. First, two levels of nesting will be explicitly modeled (caregiver and facilitator perspectives). At the third level of nesting, models may condition on a series of dummy codes capturing possible school-level effects. Second, the effect of primary interest is the test of the study condition (e.g., MFG-parent peer, MFG-community worker, comparison) by outcome trajectory (child behavioral symptoms and functioning) interaction. Third, time will be coded judiciously in accordance with a priori hypotheses about the time lags of effects of the experimental interventions. To adequately model hypothesized trajectories, higher-order polynomials can be estimated under different centering specifications, providing for multiple interpretations of the intercept and low-order trend components. Time contrasts can also be orthonormalized to reduce potential problems of multicollinearity in models specifying higher-order growth forms. Relevant trajectory variance components will also be evaluated to determine if there is variation (which would then be modeled as a function of both constant and time-varying covariates).

### Aim 2. To compare the uptake and implementation of MFGs by trained family peers and CHWs

#### Hypothesis

Given the level of training that CHWs receive prior to the study, they will evidence higher-fidelity initially, yet with training and ongoing supervision, we expect these differences to decrease over time.

To evaluate the fidelity, quality of implementation for MFGs implemented by family peers and CHWs, we will carry out a series of descriptive analyses and examine level of fidelity in four domains (described above). We will examine fidelity by year first to understand any change patterns over time, and then will combine analyses across years if no meaningful differences are found. Finally, see random-effects modeling to be used to examine differences by implementers over time.

### Aim 3. To elucidate multi-level factors that influence uptake, implementation, and youth outcomes

The overall analytic structure uses a three-level, multi-level SEM (MSEM) framework with family level variables at level 1, facilitator variables at level 2, and school variables at level 3. The analytic structure is complex, so we highlight our approach using Fig. 1 as our primary reference. We will employ mediation analysis with two-level MSEM models as explicated by Preacher, Zhang and Zyphur [119] and extended to three level models by Preacher [87]. We will use a combination of limited information estimation frameworks (focused on sub-portions of the full model) and full information estimation frameworks (focused on the entire three-level model) to strategically answer questions. At level 1, child outcomes are modeled as a function of the family predictors (see Fig. 1), taking into account the clustering at the higher levels of the model. The follow-up (6-month) measures of the mediators and the outcomes are included within the model in a classic SEM panel model with autoregressive effects, thereby linking the follow-up data to the immediate post-test data. This is an advantage of using MSEM over traditional multi-level modeling. Different centering strategies (e.g., grand mean vs. group mean) can be explored to garner various perspectives on the data. This feature of the model provides perspectives on the relative importance of paths in Fig. 1. To ascertain perspectives of the effects of school-level variables on facilitator variables, random intercept MSEM with the facilitator variables can be estimated as a function of the school variables that include dummy-coded treatment variables (MFG-parent peer vs. MFG-CHW) impacting the school mediators of readiness, leadership support and climate, which in turn, affect the facilitator-level intercepts. This modeling, or variants of it, address aim 3.

#### Integration of qualitative and quantitative analyses

We will use three different formats as described by Creswell et al. [120] for merging or converging the two datasets by bringing them together, connecting them by having one build upon the other; and embedding one dataset within the other so that one plays a supportive role for the other. Merging will occur through data triangulation in which results of quantitative and qualitative analyses are placed side by side to determine whether each provides the same answer to the same question (convergence). Results of our qualitative analyses will be connected to results from our quantitative analyses when we use the former to provide explanations for unanticipated findings produced by the latter (expansion). Finally, results of qualitative analysis can be embedded within the analysis of quantitative data by helping to provide context for outcomes (complementarity).

#### Data quality and management

The study will be overseen by local principal investigators. They will monitor the trial on a continuous basis and report to the Multiple Principal Investigators (MPIs) on a weekly basis via email, telephone or in person. Moreover, the study data will be reviewed by the National Institute of Mental Health Data and Safety Monitoring Board (DSMB) twice a year. The DSMB is established independent of the researchers involved in the study. The Board will oversee conduct of the study, particularly participant safety and data integrity. The Board has access to all the safety and data quality information collected and has the authority to stop the study if the Board believes the study is leading to unacceptable risks to participants. The study team will submit a data report to the DSMB twice yearly and upon request. The data report and other submissions to the DSMB will adhere to the established reporting format developed in consultation with National Institute of Mental Health (NIMH) collaborator(s). The report will include the major variables necessary for monitoring study participant safety, data integrity, and protocol fidelity, including study participants’ enrollment and retention. All the data reported to DSMB will be de-identified data to protect participants’ privacy. The DSMB will also review the study protocol, informed consent forms and procedures, and all relevant documents before the onset of the study, and will review and approve amendments to these documents.

The MPIs and Data Coordinating Unit (DCU) will prepare updates to the DSMB twice a year. In collaboration with DCU, the in-country data manager will organize data reports regarding preliminary process and outcome analyses (data collection at 8 weeks) and share with the MPIs. If preliminary analyses reveal harm (outcomes of child behavior are disproportionately negative), the results will be thoroughly reviewed by the MPIs and DSMB to assess whether stopping is warranted. However, no negative results have been identified in prior studies using the MFG intervention.

This study will be stopped prior to its completion if:

1. The intervention is associated with adverse effects that call into question the safety of the intervention
2. Difficulty in study participant recruitment or retention will adversely affect the ability to evaluate the study endpoints
3. Any new information becomes available during the trial that necessitates stopping the trial; or
4. Other situations occur that might warrant stopping the trial

#### Data Coordinating Unit (DCU)

This unit will be housed at Washington University in St. Louis and will be dedicated to the proposed research study. Data Coordinating Unit responsibilities include: (1) Creating a shared infrastructure that will facilitate the development and implementation of high quality study design, data collection, analysis, and development of publications across multiple projects (e.g., creation of a participant-tracking data base for scale-up study and small-scale implementation trials; assistance in creating data bases to organize data; assistance in planning and carrying out data entry and analyses of pilot data); (2) Developing core assessment battery data system (e.g., organize existing instruments; facilitate review of measures by collaborators to provide feedback); (3) Organizing consultation with on issues regarding protocol design, research methodology, measurement instruments and models, and data-analysis considerations.

#### Quality assurance plan

The MPIs and site PI are responsible at all phases of project implementation and for ensuring that the validity and integrity of the data, including study participants’ recruitment, enrollment, enrollment targets, and data collection procedures. MPIs, in collaboration with the site PI will implement quality assurance measures, including ongoing monitoring of the scale-up and pilot study activities, weekly correspondence with the local PI, and the timely reporting of any unexpected or expected adverse events that might occur during the study period. Moreover, the study will be monitored by external monitors twice a year (independent from investigators and the sponsor). The external monitors will write a report upon completing their audit, which will be shared with the local PI, MPIs, and the sponsoring institution (NIMH).

### Ethical considerations

This study poses more than minimal risk, but also has potential direct benefit for study participants. There may be psychological or privacy risks. Though highly unlikely, there is some risk for those in MFG groups of physically aggressive behavior because the study involves grouping children with behavioral challenges together:

1. It is unlikely, though possible, that intervention facilitators may feel coerced into participating. We will assure all potential study participants that their participation is voluntary. Voluntary participation will be discussed with all potential participants and they will be instructed that if they choose not to participate, it will not affect their employment status
2. A second risk is the potential loss of confidentiality. Respondents may reveal sensitive information during family groups and/or data collection. All participating groups will be encouraged to respect and protect the confidentiality of information shared during groups. All research staff and facilitators will sign confidentiality pledges. If the research team detects breach of confidentiality by research staff or facilitator, their responsibilities related to the study will be terminated.
  We will also take the utmost caution to protect the confidentiality of responses to measures. Hard copies of documents and audiotapes will be maintained in locked file cabinets of a locked office in a secure building at the International Center for Child Health and Asset Development (ICHAD) in Uganda, at the School of Public Health at University of Ghana in Ghana, and at the Department of Psychiatry at University of Nairobi in Kenya. All data will be coded with ID numbers and stored in computerized datasets. All documents and audiotapes will be transported in locked carrier bags. Participant consent forms and ID logs will be kept in two separate locked cabinets in a separate in-country location. Tracking information also will be kept on a password-protected computer. The master list will only be used to coordinate data collection and all staff will be required to receive training on both study participants protections, as well as maintaining the confidentiality of participants
3. For those in MFG groups, though highly unlikely, there may be some risk of physically challenging behavior because the program involves having children with behavioral challenges in a group setting together. The PI has conducted research studies using this intervention for over a decade and never experienced a physically aggressive incident. However, to minimize risk, both research staff and facilitators will be trained to diffuse these circumstances if they occur. The research team will make appropriate referrals to the families who need additional support
4. Among families, the most serious risk in the proposed research is any adverse outcome that may jeopardize the welfare of a participating child or family. In general, when conducting child mental health service research with children and their families, potential risks of adverse outcomes exist as a consequence of the progression of a serious mental health issue or response to a service provided. The principle investigators will be directly involved in training all research staff (including senior project coordinator, research coordinator(s), and research assistants) and facilitators to identify key indicators of any increase in physical or mental health problems among the children and families participating in the studies across the three countries. In addition to facilitators’ observations in each session, the study participants will be monitored throughout the study and data on targeted outcomes will be collected at the mid-point of the intervention as well as at 16 weeks, the end of the intervention. That will give the research team the opportunity to track potential worsening of symptoms. Research staff will be on site at least every other session, which will give facilitators the opportunity to communicate any observation of worsening symptoms. All reporting will be documented

In addition to weekly meetings with children and their caregivers, as part of the intervention over the course of 16 weeks, the research team will monitor individual study participants throughout the study and data on targeted outcomes will be collected at the mid-point of the intervention as well as at 16 weeks, the end of the intervention. That will give the research team the opportunity to track potential worsening of symptoms. There is a potential risk that a child participant’s symptoms may worsen as a consequence of the progression of a serious mental health issue or in response to a service provided. If this occurs, the participant’s study participation will be halted and the in-country research team will make the necessary referrals in coordination with the local PI. Moreover, significant impairment/deterioration will be assessed via observation (e.g., physical presentation during the study), and/or caregiver/child self-report (e.g., disclosure of suicidal ideation)

5. Participants may feel discomfort in answering some questions in the measures. They will be reminded during data collection, that they can skip any questions that they do not feel comfortable answering

## Discussion

There is an increasing interest in and use of social safety nets to achieve health outcomes for children. At the same time, there is a need to understand how psychosocial interventions can complement these economic programs for enhanced impact on mental and behavioral health. The present study protocol describes a scale-up experimental study using a mixed-methods, hybrid type II effectiveness implementation design to examine the implementation and effectiveness of an MFG family strengthening intervention, aimed at improving child disruptive behavioral challenges in Uganda. The study will also examine multi-level factors that influence uptake, implementation, sustainment, and youth outcomes.

We hypothesize that children participating in MFG will display significantly reduced behavioral difficulties and increased functioning over time compared to those involved in the comparison condition. We expect that parent peers will evidence significantly more success engaging families to attend MFG sessions, and thus children in the MFG-parent-peer-delivered condition will evidence the greatest improvement relative to the other two study conditions. We expect that, given the level of professional training that CHWs have received prior to the study, they will evidence higher protocol fidelity than parent peers, at least initially. Yet, with training and ongoing supervision for parent peers, we expect these differences across groups to decrease over time. Hence, the study findings will yield important insights on the effectiveness of MFG intervention in reducing child behavioral challenges while also exploring contextual factors that may influence MFG implementation, sustainability, and impact.

We do not anticipate any major threats to study implementation though we recognize potential concerns and have adapted accordingly. We have a stringent retention plan for attendance at the MFG sessions. We expect to achieve our enrollment and retention goals based on our current and previous studies among school going children in the same study area. However, we are aware that as children grow, they are more likely to leave school, and some may migrate to work before completing the intervention. As such, should our study participants’ recruitment, enrollment or retention rates deviate from anticipated goals, we will consult with the research and implementing team to adjust our outreach activities accordingly.

Despite the potential limitations identified above, the proposed project is highly innovative in multiple ways. *First*, the study has been designed to align with the priorities and preferences of Ugandan policy-makers in the MOH and MOE. *Second*, building on existing child and family focused EBPs, [38, 55, 56, 65–71] this is the first investigation of a family centered approach of DBD interventions provided within the lower primary school context (before children drop out of school), with the aim of reaching a large child population with mental health service needs in SSA countries. If successful, the potential public health impact is high. *Third*, our study draws upon an EBP package that has already been tested with delivery by parent peers and community facilitators and will take advantage of the advancing science behind task-shifting, defined as “engaging non-specialists in the provision of effective psychosocial treatments under the supervision of mental health specialists” [74]. The task-shifting approach to supporting lay workers and peers already exists in health and education systems, and thus is promising as a cost-efficient and feasible model for SSA countries. This study tests two task-shifting approaches (task-shifting intervention skills to CHWs and parent peers). It is possible that testing a task-shifting, large-scale implementation strategy in low-resource SSA settings can facilitate “reverse innovation.” Effective services and implementation strategies identified in developing countries may facilitate new innovations to address similar CAMH disparities in US populations or in other developed countries. *Fourth*, the collaborative approach is innovative as it includes capacity building and collaboration among regional health and education policy stakeholders, experts from multiple disciplines, and community members. The approach will help stimulate application of science-based policy decisions, enhance local community leaders’ and members’ participation in resolving CAMH service gaps, and increase the chance for program sustainment, dissemination, and policy change. *Finally*, several methods employed in this study are innovative. This study blends in clinical effectiveness and implementation research methods [81], and incorporates strategies (utilizing PRISM) to investigate scale-up-related mechanisms and implementation research questions, which can have more rapid translational gains, inform more effective implementation strategies, and provide more useful information for decision-makers. In summary, this study has great potential to address global child mental health needs.

In sum, this study has been carefully designed with contextual issues at the forefront. An in-country study team consisting of academic researchers, NGOs, government officials, and community/cultural leaders) will lead MFG systemic adaptation efforts so that, if findings warrant, there will be an increased chance that the intervention can be rolled out easily to an expanded range of child-serving settings and implemented by existing workforces. Our partnership with the Ministries and Departments of Health and Education also increases the likelihood that findings will influence large-scale roll outs, especially since the MFGs in the US and SA have already been delivered by lay counselors, family peers, and trained community workers in schools and public health clinics.

### Trial status

At the time of manuscript submission, the trial had received DSMB and Institutional Review Board (IRB) approvals and was in the process of study participant recruitment and baseline assessment. The study is ongoing.

## Additional file

Additional file 1: Standard Protocol Items: Recommendations for Interventional Trials (SPIRIT) 2013 Checklist. (DOC 141 kb)

## Abbreviations

CFI: Comparative Fit Index
CHAID: Chi-squared Automatic Interaction Detector
CHAMP: Collaborative HIV prevention and Adolescent Mental Health Project
CHWs: Community health workers
DBDs: Disruptive behavioral disorders
DCU: Data Coordinating Unit
DSMB: Data and Safety Monitoring Board
EBPs: Evidence-based practices
KSAT: Knowledge and Skills Assessment Test
LMICs: Low and middle-income countries
MFG: Multiple Family Group
MOE: Ministry of Education
MOH: Ministry of Health
MPIs: Multiple Principal Investigators
MSEM: Multi-level structural equation modeling
NIMH: National Institute of Mental Health
PI: Principle investigator
PRISM: Practical, Robust Implementation and Sustainability Model
PTA: Parent Teacher Association
RE-AIM: Reach-Effectiveness-Adoption-Implementation-Maintenance
RMR: Root Mean Square Residual
RMSEA: Root Mean Square Error of Approximation
SAMHSA: Substance Abuse and Mental Health Services Administration
SSA: Sub-Saharan Africa
WHO: The World Health Organization

## References

[1] Roberts M, Mogan C, Asare JB (2014). An overview of Ghana’s mental health system: results from an assessment using the World Health Organization’s assessment instrument for mental health systems (WHO-AIMS). Int J Ment Health Syst.

[2] Kieling C, Baker-Henningham H, Belfer M, Conti G, Ertem I, Omigbodun O (2011). Child and adolescent mental health worldwide: evidence for action. Lancet.

[3] World Health Organization, World Psychiatric Association, International Association for Child, Adolescent Psychiatry, Allied Professions. Atlas: child and adolescent mental health resources, global concerns, implications for the future. Geneva: World Health Organization; 2005.

[4] Giel R, Harding TW (1976). Psychiatric priorities in developing countries. Br J Psychiatry.

[5] Nalugya J (2004). Depression amongst secondary school adolescents in Mukono district, Uganda. Doctoral dissertation, dissertation.

[6] Loeber R, Green SM, Lahey BB, Frick PJ, McBurnett K (2000). Findings on disruptive behavior disorders from the first decade of the developmental trends study. Clin Child Fam Psychol Rev.

[7] Loeber R, Burke JD, Lahey BB, Winters A, Zera M (2000). Oppositional defiant and conduct disorder: a review of the past 10 years, part I. J Am Acad Child Adolesc Psychiatry.

[8] Burke JD, Loeber R, Birmaher B (2002). Oppositional defiant disorder and conduct disorder: a review of the past 10 years, part II. J Am Acad Child Adolesc Psychiatry.

[9] Belfer ML (2008). Child and adolescent mental disorders: the magnitude of the problem across the globe. J Child Psychol Psychiatry.

[10] Ssewamala FM, Nabunya P, Ilic V, Mukasa MN, Damulira C (2015). Relationship between family economic resources, psychosocial well-being, and educational preferences of AIDS-orphaned children in southern Uganda: baseline findings. Glob Soc Welf.

[11] Ssewamala FM, Ismayilova L, McKay M, Sperber E, Bannon W, Alicea S (2010). Gender and the effects of an economic empowerment program on attitudes toward sexual risk-taking among AIDS-orphaned adolescent youth in Uganda. J Adolesc Health.

[12] Curley J, Ssewamala FM, Nabunya P, Ilic V, Han CK (2014). Child development accounts (CDAs): an asset-building strategy to empower girls in Uganda. Int Soc Work.

[13] Nabunya P, Ssewamala FM (2014). The effects of parental loss on the psychosocial wellbeing of AIDS-orphaned children living in AIDS-impacted communities: does gender matter?. Child Youth Serv Rev.

[14] Hawkins JD, Catalano RF, Miller JY (1992). Risk and protective factors for alcohol and other drug problems in adolescence and early adulthood: implications for substance abuse prevention. Psychol Bull.

[15] Carlson CL, Tamm L, Hogan AE, Quay HE, Hogan AE (1999). The child with oppositional defiant disorder and conduct disorders in the family. Handbook of disruptive behavior disorders.

[16] Lendingham JE, Quay HE, Hogan AE (1999). Children and adolescents with oppositional defiant disorder and conduct disorder in the community: experiences at school and with peer. Handbook of disruptive behavior disorders.

[17] Bellis MA, Lowey H, Leckenby N, Hughes K, Harrison D (2013). Adverse childhood experiences: retrospective study to determine their impact on adult health behaviors and health outcomes in a UK population. Public Health.

[18] Washburn J, Teplin L, Voss L, Simon C, Abram K, McClelland G (2008). Psychiatric disorders among detained youths: a comparison of youths processed in juvenile court and adult criminal court. Psychiatr Serv.

[19] Farrington DP (1995). The development of offending and antisocial behaviour from childhood: key findings from the Cambridge study in delinquent development. J Child Psychol Psychiatry.

[20] Kazdin AE (1995). Conduct disorders in childhood and adolescence.

[21] Apkan MU, Ojinnaka NC, Ekanem E (2010). Behavioral problems among schoolchildren in Nigeria. South Afr J Psychiatry.

[22] Ashenafi Y, Kebede D, Desta M, Alem A (2001). Prevalence of mental and behavioural disorders in Ethiopia. East Afr Med J.

[23] Cortina MA, Sodha A, Fazel M, Ramchandani PG (2012). Prevalence of child mental health problems in sub-Saharan Africa: a systematic review. Arch Pediatr Adolesc Med.

[24] Liang H, Flisher AJ, Chalton DO (2002). Mental and physical health of out of schoolchildren in a South African township. Eur Child Adolesc Psychiatry.

[25] UNICEF (2015). State of the world’s children 2015 country statistical tables: Uganda statistics.

[26] Population Reference Bureau (2009). World population data sheet.

[27] World Health Organization (2009). Country profile of environmental burden of disease: Uganda.

[28] Koenig MA, Lutalo T, Zhao F, Nalugoda F, Wabwire-Mangen F, Kiwanuka N (2003). Domestic violence in rural Uganda: evidence from a community-based study. Bull World Health Organ.

[29] Naker D (2005). Violence against children: The voices of Uganda children and adults.

[30] Ovuga E, Boardman J, Wasserman D (2005). The prevalence of depression in two districts of Uganda. Soc Psychiatr Epidemiol.

[31] Brownstein JN, Bone LR, Dennison CR, Hill MN, Kim MT, Levine DM (2005). Community health workers as interventionists in the prevention and control of heart disease and stroke. Am J Prev Med.

[32] WHO: Uganda Country Profile. 2013. http://www.who.int/countries/uga/en/. Accessed 30 June 2015.

[33] Loeber R, Farrington DP, Stouthamer-Loeber M, Van Kammen WB (1998). Antisocial behavior and mental health problems: explanatory factors in childhood and adolescence.

[34] Dishion TJ, French DC, Patterson GR, Cicchetti D, Cohen DJ (1995). The development and ecology of antisocial behavior. Developmental psychopathology, vol. 2: risk, disorder, and adaptation.

[35] Murray CJ, Lopez AD (2013). Measuring the global burden of disease. N Engl J Med.

[36] O’Callaghan P, McMullen J, Shannon C, Rafferty H, Black A (2013). A randomized controlled trial of trauma-focused cognitive behavioral therapy for sexually exploited, war-affected Congolese girls. J Am Acad Child Adolesc Psychiatry.

[37] Bolton P, Bass J, Betancourt T, Speelman L, Onyango G, Clougherty KF (2007). Interventions for depression symptoms among adolescent survivors of war and displacement in northern Uganda: a randomized controlled trial. JAMA.

[38] Bell CC, Bhana A, Petersen I, McKay M, Gibbons R, Bannon W, Amatya A (2008). Building protective factors to offset sexually risky behaviors among black youths: a randomized control trial. J Natl Med Assoc.

[39] McKay M, Alicea S, Elwyn L, McClain Z, Parker G, Small L, Mellins C (2014). Addressing the need for theory-driven programs capable of impacting urban children’s health, mental health, and prevention needs: CHAMP and CHAMP+, evidence-informed, family-based interventions to address HIV risk and care. J Child Adolesc Psychol.

[40] Bhana A, Mellins C, Petersen I, Alicea S, Myeza N, Holst H, Abrams E, Johns S, Chhagan M, Nestadt D, McKay M (2013). The VUKA Family Program: piloting a family-based psychosocial intervention to promote health and mental health among HIV+ early adolescents in South Africa. AIDS Care.

[41] Huang KY, Calzada E, Kamboukos D, Rhule D, Sharma KC, Cheng S, Brotman LM (2014). Applying public health frameworks to advance the prevention of mental health problems in Asian American children. Asian Am J Psychol.

[42] WHO: Mental Health Action Plan 2013–2020. 2013. http://apps.who.int/iris/bitstream/10665/89966/1/9789241506021_eng.pdf. Accessed 29 June 2015.

[43] Feldstein AC, Glasgow RE (2008). A practical, robust implementation and sustainability model (PRISM) for integrating research findings into practice. Jt Comm J Qual Patient Saf.

[44] Masseli D, Lys JA, Schmid J (2005). Improving impacts of research partnerships.

[45] Aikins ADG, Arhinful DK, Pitchforth E, Ogedegbe G, Allotey P, Agyemang C (2012). Establishing and sustaining research partnerships in Africa: a case study of the UK-Africa Academic Partnership on Chronic Disease. Glob Health.

[46] Kazdin AE, Whitley MK (2003). Treatment of parental stress to enhance therapeutic change among children referred for aggressive and antisocial behavior. J Consult Clin Psychol.

[47] Keiley MK (2002). The development and implementation of an affect regulation and attachment intervention for incarcerated adolescents and their parents. Fam J.

[48] Kumpfer KL, Alvarado R, Smith P, Bellamy N (2002). Cultural sensitivity and adaptation in family-based prevention interventions. Prev Sci.

[49] Wahler RG, Dumas JE (1989). Attentional problems in dysfunctional mother-child interactions: an interbehavior model. Psychol Bull.

[50] Webster-Stratton C (1985). Case studies and clinical replication series: predictors of treatment outcome in parent training for conduct disordered children. Behav Ther.

[51] Webster-Stratton C, Hammond M (1990). Predictors of treatment outcome in parent training for families with conduct problem children. Behav Ther.

[52] Sexton TL, Alexander JF (2002). Family-based empirically supported interventions. Couns Psychol.

[53] Farmer E, Lipscombe J, Moyers S (2002). Foster care strain and its impact on parenting and placement outcomes for adolescents. Br J Soc Work.

[54] Cottrell D, Boston P (2002). Practitioner review: the effectiveness of systemic family therapy for children and adolescents. J Child Psychol Psychiatry.

[55] Gopalan G, Franco L, Dean-Assael K, McGuire-Schwartz M, Chacko A, McKay M (2014). Statewide implementation of the 4Rs and Ss for strengthening families. J Evid Based Soc Work.

[56] Gopalan G, Chacko A, Franco L, Dean-Assael K, Rotko L, Marcus S (2014). Multiple family group service delivery model for youth with disruptive behaviors: child outcomes at 6 month follow-up. J Child Fam Stud.

[57] Chacko A, Gopalan G, Franco L, Dean-Assael K, Jackson J, Marcus S (2015). Multiple family group service model for children with disruptive behavior disorders: child outcomes at post-treatment. J Emot Behav Disord.

[58] McKay MM, Gopalan G, Franco L, Dean-Assael K, Chacko A, Jackson J (2011). A collaboratively designed child mental health service model: multiple family groups for urban children with conduct difficulties. Res Soc Work Pract.

[59] McKay MM, Gonzales J, Quintana E, Kim L, Abdul-Adil J. Multiple family groups: an alternative for reducing disruptive behavioral difficulties of urban children. Res Soc Work Pract. 1999;9(5):593–607.

[60] McKay M, Jensen P, Hoagwood K, Jensen P, McKay M, Olin S, the CHAMP Collaborative Board (2010). Collaborative child mental health services research: theoretical perspectives and practical guidelines. Collaborative research to improve child mental health services.

[61] Bhana A, McKay MM, Mellins C, Petersen I, Bell C (2010). Family-based HIV prevention and intervention services for youth living in poverty-affected contexts: the CHAMP model of collaborative, evidence-informed programme development. J Int AIDS Soc.

[62] Baptiste DR, Bhana A, Petersen I, McKay M, Voisin D, Bell C, Martinez DD (2006). Community collaborative youth-focused HIV/AIDS prevention in South Africa and Trinidad. J Pediatr Psychol.

[63] Bhana A, Petersen I, Mason A, Mahintsho Z, Bell C, McKay M (2004). Children and youth at risk: adaptation and pilot study of the CHAMP (AmaQhawe) programme in South African. Afr J AIDS Res.

[64] Kinyanda E, Woodburn P, Tugumisirize J, Kagugube J, Ndyanabangi S, Patel V (2011). Poverty, life events and the risk for depression in Uganda. Soc Psychiatry Psychiatr Epidemiol.

[65] Ssewamala FM, Alicea S, Bannon WM, Ismayilova L (2008). A novel economic intervention to reduce HIV risks among school-going AIDS orphans in rural Uganda. J Adolesc Health.

[66] Ssewamala FM, Ismayilova L (2009). Integrating children’s savings accounts in the care and support of orphaned adolescents in rural Uganda. Soc Serv Rev.

[67] Ssewamala FM, Karimli L, Chang-Keun H, Ismayilova L (2010). Social capital, savings, and educational performance of orphaned adolescents in sub-Saharan Africa. Child Youth Serv Rev.

[68] Ssewamala FM, Han CK, Neilands TB, Ismayilova L, Sperber E (2010). Effect of economic assets on sexual risk-taking intentions among orphaned adolescents in Uganda. Am J Public Health.

[69] Ssewamala FM, Han CK, Neilands TB (2009). Asset ownership and health and mental health functioning among AIDS-orphaned adolescents: findings from a randomized clinical trial in rural Uganda. Soc Sci Med.

[70] Ssewamala FM, Neilands TB, Waldfogel J, Ismayilova L (2012). The impact of a comprehensive microfinance intervention on depression levels of AIDS-orphaned children in Uganda. J Adolesc Health.

[71] Han CK, Ssewamala FM, Wang JSH (2013). Family economic empowerment and mental health among AIDS-affected children living in AIDS-impacted communities: evidence from a randomized evaluation in southwestern Uganda. J Epidemiol Community Health.

[72] Weisz JR, Jensen AL (2001). Child and adolescent psychotherapy in research and practice contexts: review of the evidence and suggestions for improving the field. Eur Child Adolesc Psychiatry..

[73] Hoagwood KE, Burns BJ, Weisz JR, Burns BJ, Hoagwood K (2002). A profitable conjunction: from science to service in children’s mental health. Community treatment for youth: evidence-based interventions for youth with severe emotional and emotional disorders.

[74] Petersen I, Mason A, Bhana A, Bell C, McKay M (2006). Mediating social representations using targeted micro media in the form of a cartoon narrative in the context of HIV/AIDS: the AmaQhawe Family Project (CHAMP) in South Africa. J Health Psychol.

[75] Mellins CA, Nestadt D, Bhana A, Petersen I, Abrams EJ, Alicea S (2014). Adapting evidence-based interventions to meet the needs of adolescents growing up with HIV in South Africa: the VUKA case example. Glob Soc Welf..

[76] Hirschhorn LR, Ojikutu B, Rodriguez W (2007). Research for change: using implementation research to strengthen HIV care and treatment scale-up in resource-limited settings. J Infect Dis.

[77] Schackman BR (2010). Implementation science for the prevention and treatment of HIV/AIDS. J Acquir Immune Defic Syndr Hum Retrovirol.

[78] Kisia J, Nelima F, Otieno DO, Kiilu K, Emmanuel W, Sohani S (2012). Factors associated with utilization of community health workers in improving access to malaria treatment among children in Kenya. Malar J.

[79] Wittkowski A, Gardner PL, Bunton P, Edge D (2014). Culturally determined risk factors for postnatal depression in sub-Saharan Africa: a mixed method systematic review. J Affect Disord.

[80] McKay M, Paikoff R (2007). Community collaborative partnerships: the foundation for HIV prevention research efforts in the United States and internationally.

[81] Curran GM, Bauer M, Mittman B, Pyne JM, Stetler C (2012). Effectiveness-implementation hybrid designs: combining elements of clinical effectiveness and implementation research to enhance public health impact. Med Care.

[82] Uganda AIDS Commission. HIV and AIDS Uganda country progress report. http://www.unaids.org/sites/default/files/country/documents/UGA_narrative_report_2014.pdf. Accessed 29 Mar 2015.

[83] Andridge RR, Little RJA (2010). A Review of hot deck imputation for survey non-response. Int Stat Rev.

[84] Briggs A, Clark T, Wolstenholme J, Clarke P (2003). Missing.... presumed at random: cost-analysis of incomplete data. Health Econ.

[85] Ono M, Miller HP. Income nonresponses in the current population survey. In Proceedings of the Social Statistics Section, American Statistical Association. 1969. p. 277–288.

[86] U.S. Bureau of the Census. Tech rep. Vol. 63. U.S. Government Printing Office; 2002. Technical paper.

[87] Preacher KJ (2011). Multilevel SEM strategies for evaluating mediation in three-level data. Multivar Behav Res.

[88] Hox JJ, Maas CJM, Brinkhuis MJS (2010). The effect of estimation method and sample size in multilevel structural equation modeling. Statistica Neerlandica.

[89] Wolf EJ, Harrington KM, Clark SL, Miller MW. Sample size requirements for structural equation models: an evaluation of power, bias, and solution propriety. Educ Psychol Meas. 2013;76(6):913–34. 10.1177/0013164413495237. 10.1177/0013164413495237 PMC4334479 25705052

[90] Hox JJ. Multilevel regression and multilevel structural equation modeling. The Oxford handbook of quantitative methods in psychology: Vol. 2: Statistical analysis.:Oxford University Press, 2013–03-21. Oxford Handbooks Online. 2013–10-01. Date Accessed 4 June 2018.

[91] Gwadz M, Rotheram-Borus MJ. Tracking high-risk adolescents longitudinally. AIDS Educ Prev. 1992;Suppl:69–82. 1389872

[92] Gibbons RD, Hedeker D, DuToit S (2010). Advances in analysis of longitudinal data. Annu Rev Clin Psychol.

[93] Hedeker D, Gibbons RD, Waternaux C (1999). Sample size estimation for longitudinal designs with attrition: comparing time-related contrasts between two groups. J Educ Behav Stat.

[94] Roy A, Bhaumik DK, Aryal S, Gibbons RD (2007). Sample size determination for hierarchical longitudinal designs with differential attrition rates. Biometrics.

[95] Bhaumik DK, Roy A, Aryal S, Hur K, Duan N, Normand SLT (2008). Sample size determination for studies with repeated continuous outcomes. Psychiatr Ann.

[96] Hedeker D, Gibbons RD (1996). MIXOR: a computer program for mixed-effects ordinal regression analysis. Comput Methods Prog Biomed.

[97] Donner A, Birkett N, Buck C (1981). Randomization by cluster: sample size requirements and analysis. Am J Epidemiol.

[98] Tolan PH, McKay M (1996). Preventing serious antisocial behavior in inner-city children. Fam Relat.

[99] Hayes R, Moulton L (2009). Cluster randomised trials.

[100] Glasgow RE, Vogt TM, Boles SM (1999). Evaluating the public health impact of health promotion interventions: the RE-AIM framework. Am J Public Health.

[101] Glasgow RE, McKay HG, Piette JD, Reynolds KD (2001). The RE-AIM framework for evaluating interventions: what can it tell us about approaches to chronic illness management?. Patient Educ Couns.

[102] Schoenwald SK, Garland AF, Chapman JE, Frazier SL, Sheidow AJ, Southam-Gerow MA (2011). Toward the effective and efficient measurement of implementation fidelity. Admin Pol Ment Health.

[103] Schoenwald SK, Henggeler SW, Brondino MJ, Rowland MD (2000). Multisystemic therapy: monitoring treatment fidelity. Fam Process.

[104] Creswell JW (2013). Research design: qualitative, quantitative, and mixed methods approach.

[105] Palinkas LA, Holloway IW, Rice E, Fuentes D, Wu Q, Chamberlain P (2011). Social networks and implementation of evidence-based practices in public youth-serving systems: a mixed methods study. Implement Sci.

[106] Palinkas LA, Weisz JR, Chorpita B, Levine B, Garland A, Hoagwood KE, Landsverk J (2016). Use of evidence-based treatments for youth mental health subsequent to a randomized controlled effectiveness trial: a qualitative study. Psychiatr Serv.

[107] NVivo qualitative data analysis software; QSR International Pty Ltd. Version 10. 2012.

[108] Palinkas LA, Aarons GA, Horwitz S, Chamberlain P, Hurlburt M, Landsverk J (2011). Mixed method designs in implementation research. Admin Pol Ment Health.

[109] Willms DG, Best AJ, Taylor DW, Gilbert JR, Wilson DMC, Lindsay EA (1992). A systematic approach for using qualitative methods in primary prevention research. Med Anthropol Q.

[110] Miles MB, Huberman AM (1994). Qualitative data analysis: an expanded sourcebook.

[111] Strauss A, Corbin J (1998). Basics of qualitative research: techniques and procedures for developing grounded theory.

[112] Boyatzis R (1998). Transforming qualitative information: thematic analysis and code development.

[113] Costello AB (2009). Getting the most from your analysis. Pract Assess Res Eval.

[114] Biggs D, de Ville B, Suen B (1991). A method of choosing multiway partitions for classification and decision trees. J Appl Stat.

[115] Wheaton B, Muthen B, Alwin D, Summers G (1977). Assessing reliability and stability in panel models. Sociol Methodol.

[116] Tabachnick BG, Fidell LS (2007). Using multivariate statistics.

[117] Hu LT, Bentler PM (1999). Cutoff criteria for fit indexes in covariance structure analysis: conventional criteria versus new alternatives. Struct Equ Model.

[118] Hooper D, Coughlan J, Mullen MR (2008). Structural equation modelling: guidelines for determining model fit. Electron J Bus Res Methods.

[119] Preacher KJ, Zhang Z, Zyphur MJ (2011). Alternative methods for assessing mediation in multilevel data: the advantages of multilevel SEM. Struct Equ Model.

[120] Creswell JW, Klassen AC, Plano Clark VL, Smith KC. Best practices for mixed methods research in the health sciences. https://www2.jabsom.hawaii.edu/native/docs/tsudocs/Best_Practices_for_Mixed_Methods_Research_Aug2011.pdf. Accessed 30 July 2015.

[121] Pelham WE, Fabiano GA, Massetti GM (2005). Evidence-based assessment of attention deficit hyperactivity disorder in children and adolescents. J Clin Child Adolesc Psychol.

[122] Waschbusch DA, Willoughby MT (2008). Parent and teacher ratings on the IOWA Conners Rating Scale. J Psychopathol Behav Assess.

[123] Fabiano GA, Pelham WE, Waschbusch DA, Gnagy EM, Lahey BB, Chronis AM (2006). A practical measure of impairment: psychometric properties of the Impairment Rating Scale in samples of children with attention deficit hyperactivity disorder and two school-based samples. J Clin Child Adolesc Psychol..

[124] Elgar FJ, Waschbusch DA, Dadds MR, Sigvaldason N (2007). Development and validation of a short form of the Alabama Parenting Questionnaire. J Child Fam Stud.

[125] Abidin RR (1990). Parenting Stress Index-short form.

[126] Derogatis LR, Fitzpatrick M, Maruish ME (2004). The SCL-90-R, the Brief Symptom Inventory (BSI), and the BSI-18. The use of psychological testing for treatment planning and outcomes assessment: instruments for adults.

[127] Goodman R, Ford T, Simmons H, Gatward R, Meltzer H (2000). Using the Strengths and Difficulties Questionnaire (SDQ) to screen for child psychiatric disorders in a community sample. Br J Psychiatry.

[128] Huebner ES, Laughlin JE, Ash C, Gilman R (1998). Further validation of the Multidimensional Students’ Life Satisfaction Scale. J Psychoeduc Assess.

[129] Kovacs M (1992). Child Depression Inventory.

[130] Fitts WH, Warren WL (1996). Tennessee Self-Concept Scale: TSCS-2.

[131] McKay M, Block M, Mellins C, Traube DE, Brackis-Cott E, Minott D (2017). Adapting a family-based HIV prevention program for HIV-infected preadolescents and their families: youth, families and health care providers coming together to address complex needs. Soc Work Ment Health.

[132] Luke DA, Calhoun A, Robichaux CB, Elliott MB, Moreland-Russell S. The program sustainability assessment tool: a new instrument for public health programs. Prev Chronic Dis. 2014;11:E12. 10.5888/pcd11.130184 PMC3900326 24456645

